# Unintentional Diagnosis of Uterus Didelphys in a Primigravida During Emergency Cesarean Section: A Case Report

**DOI:** 10.7759/cureus.113472

**Published:** 2026-07-27

**Authors:** Dil Sahi Roka, Priyanka Gharti Magar, Jie Hou, Lijing Liu

**Affiliations:** 1 Obstetrics and Gynecology, The Third Affiliated Hospital of Qiqihar Medical University, Qiqihar, CHN; 2 Nursing, Thompson Health Care, Sydney, AUS

**Keywords:** cesarean section (cs), congenital uterine anomaly, didelphys uterus, müllerian duct anomalies, pg primigravida

## Abstract

Uterus didelphys is a rare congenital Müllerian duct anomaly defined by the presence of two uteri, typically with two cervices and occasionally with a vaginal septum. Although in many cases it is asymptomatic, it is linked to several complications, including miscarriage, premature birth, malpresentation, and an increased rate of cesarean section. We report a 19-year-old primigravida incidentally diagnosed with uterus didelphys at emergency cesarean section for fetal distress at 38 weeks. Uterus didelphys is one of the rare Müllerian duct anomalies and is often missed on routine antenatal ultrasonography. Intraoperatively, a non-gravid right uterine horn and a gravid left uterine horn were noted. A healthy male infant of 2.7 kg was delivered. The postoperative period was uneventful and highlights the importance of careful antenatal evaluation, proper counseling, and multidisciplinary team involvement in future pregnancies.

## Introduction

Congenital anomalies of the female reproductive tract occur as a result of abnormal development, fusion, or resorption of the Müllerian ducts during embryogenesis. They are present in approximately 0.5-6.6% of women in the general population [1]. Uterus didelphys results from a complete failure of fusion of the Müllerian ducts, resulting in two uterine cavities and typically two cervices, occasionally with a vaginal septum, and it belongs to class 3 of the American Society for Reproductive Medicine (ASRM) classification [2]. Although the condition may be associated with normal fertility, it is related to miscarriage, preterm labor, malpresentation, and increased incidence of cesarean section [3,4]. Diagnosis is typically confirmed through ultrasound or MRI [5]. During antenatal scans, the focus is primarily on the fetus rather than uterine anatomy, and a large gravid uterus sometimes obscures the view of a small non-gravid uterus, especially in the mid and late trimesters, making the anomaly difficult to detect. In some cases, as in the present case, the anomaly may be discovered unexpectedly during emergency cesarean section. This highlights the importance of being prepared for rare conditions during labor and of obtaining a detailed ultrasound scan of the fetus as well as uterine anatomy at antenatal visits.

## Case presentation

A 19-year-old primigravida at 38 weeks of gestation presented with complaints of regular labor pain and reduced fetal movements to the emergency room for six hours. Her menarche was at the age of 12 years, and she had regular menstrual cycles lasting four to seven days every 28-30 days. She was asymptomatic and had never undergone an ultrasound examination previously for other indications. She was unaware of a similar problem in her family and had no history of any surgeries. The BMI was within the normal range, and she denied an anomaly scan as she lived far from the city in a remote area. She had attended routine antenatal visits in a local health facility with no ultrasound facility available, and her pregnancy had been deemed uneventful. The previous two obstetric ultrasound scans in the second and third trimesters reportedly showed a single intrauterine pregnancy with no structural uterine abnormalities.

On admission, the patient was conscious and hemodynamically stable. Her blood pressure was recorded as 118 over 76 mmHg, pulse rate was 86 per minute, and temperature was within normal limits. Obstetric examination revealed a fundal height of 38 weeks' gestation and cephalic presentation. Fetal heart monitoring showed a baseline fetal heart rate of 100 beats per minute with repeated decelerations on cardiotocography, which was suggestive of fetal distress.

Lab investigations were within normal limits, and the serology tests were non-reactive (Table 1) [6]. A blood coagulation profile was done as part of preoperative investigations for surgery. Renal function tests were done as part of routine preoperative investigations, but not due to possible related renal anomalies in this case.

**Table 1:** Laboratory investigations profile RBC: red blood cell count, WBC: white blood cell count, PT: prothrombin time, aPTT: activated partial thromboplastin time, INR: international normalized ratio

Parameter	Result	References	Unit
Haemoglobin	12.8	9.5-15	g/dL
RBC	3.2	2.71-4.43	×10^6^/mm^3^
WBC	8.8	5.9-16.9	×10^3^/mm^3^
Platelets	155	146-429	×10^9^/L
Urea	32	15-45	mg/dL
Creatinine	0.6	0.4-0.9	mg/dL
Sodium	142	130-148	mEq/L
Potassium	4.3	3.3-5.1	mEq/L
PT	0.90	9.6-12.9	sec
aPTT	29.2	24.7-35	sec
INR	0.88	0.80-0.94	-

A perivaginal examination revealed two cervixes. The right cervix was closed, the left cervical dilation was 3 cm, and membranes were intact. Due to the persisting fetal bradycardia and suspicions of fetal compromise, an emergency cesarean section was performed.

**Figure 1:**
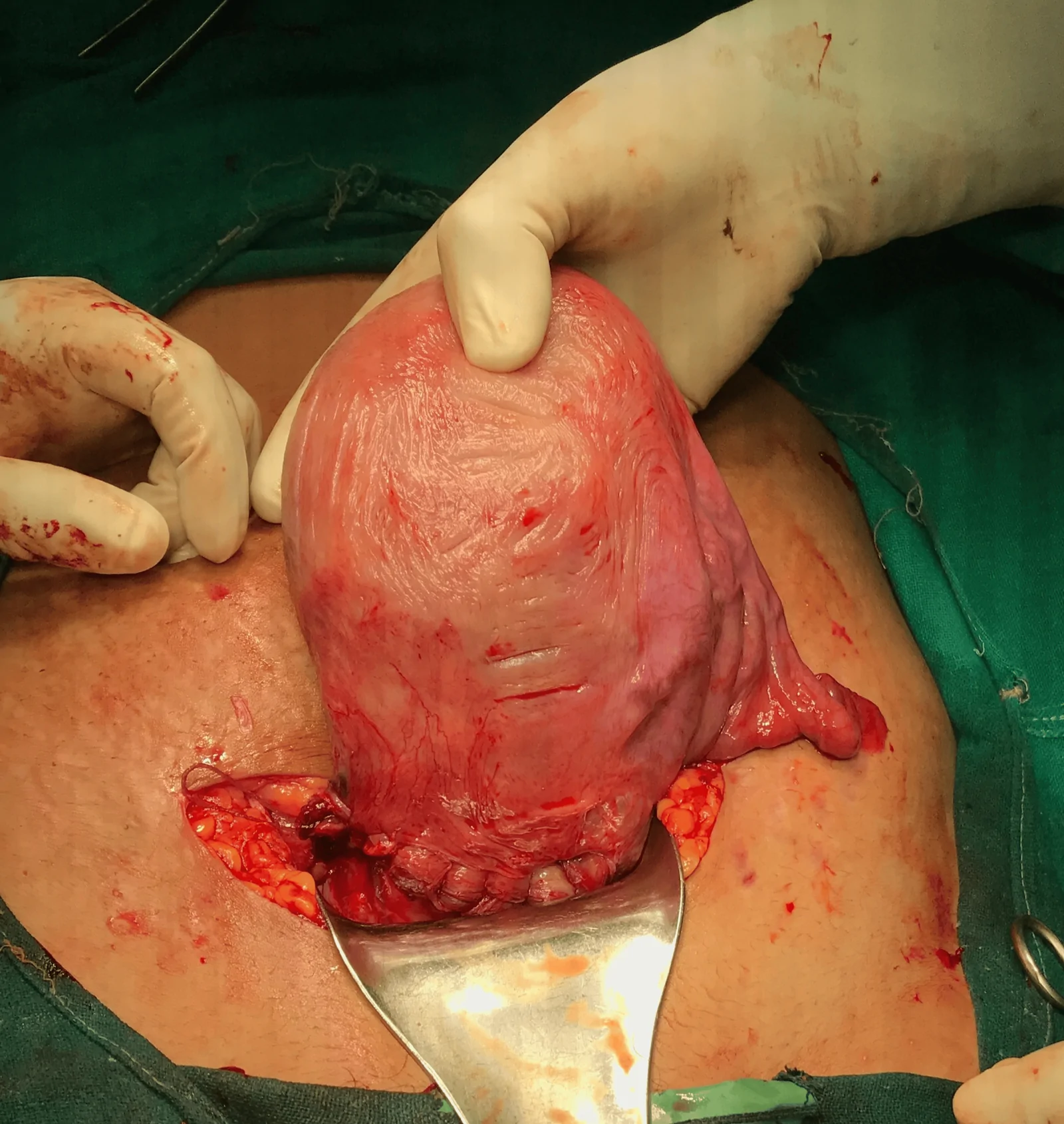
Image showing single uterus immediately after closing the left horn incision

**Figure 2:**
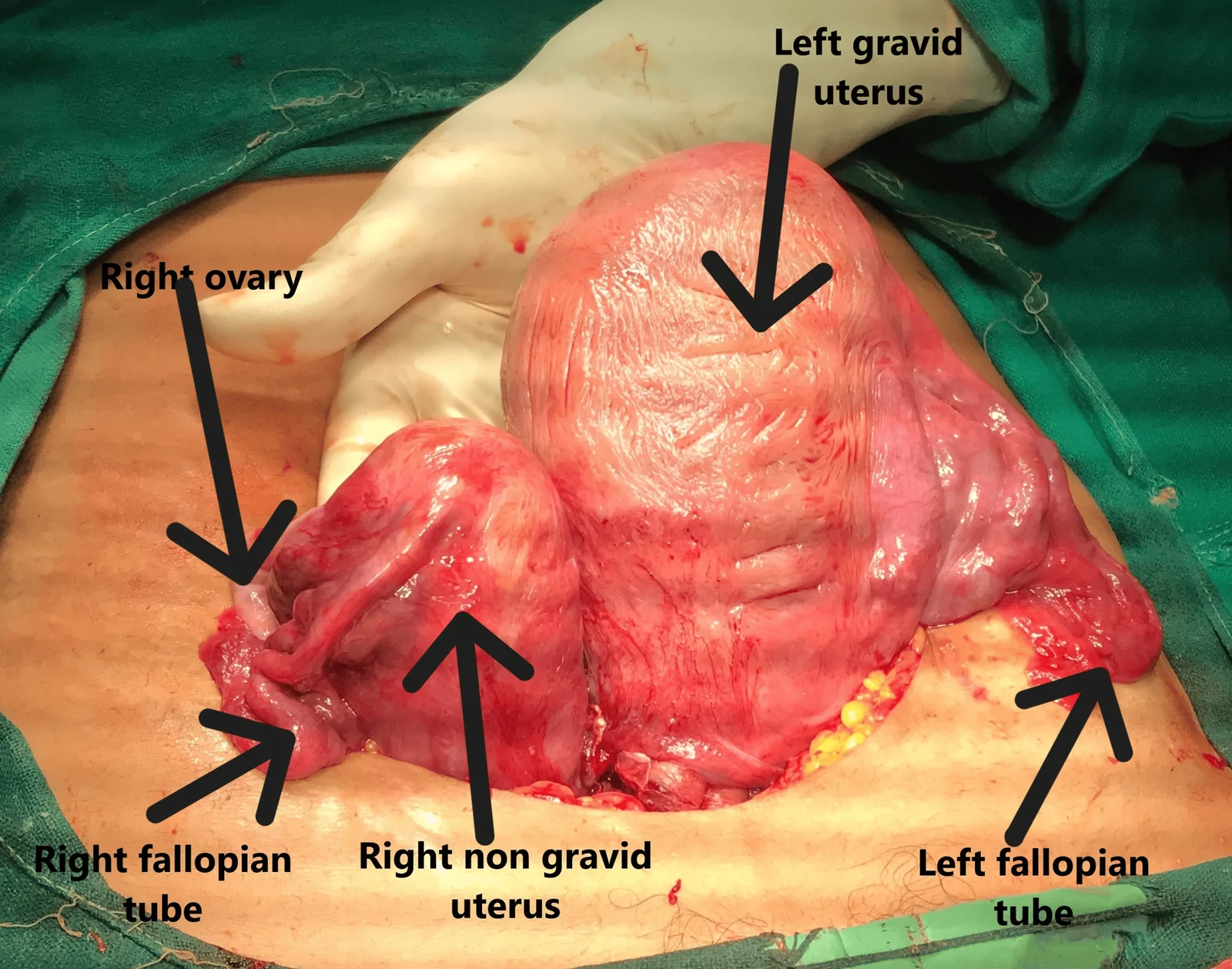
Image showing two separate uteri, each with its own fallopian tube and ovary

During the operation, an unusual anatomy of the uterus was discerned. Initially, the anatomy appeared unusual, as we saw a single uterus with unilateral agenesis of fallopian tubes and ovaries (Figure 1). Further exploration showed two completely separate uterine cavities, which is consistent with uterus didelphys. The pregnancy was located in the left uterine horn, and the right side of the uterus was smaller and not gravid (Figure 2). Each of the uterine horns had its own cervix, and the fallopian tubes, ovaries, and round ligament were attached laterally to each uterine horn. The broad ligament was attached to both horns, and the placenta was implanted fundoposterior in the left uterus. Finally, the diagnosis of uterus didelphys was made incidentally during surgery.

A live male infant of 2.7 kg was delivered with Apgar scores of 8 and 9 at 1 and five minutes, respectively, with the uterine incision made in the left uterine horn carefully without extending to the opposite horn. The placenta was delivered using controlled cord traction, and the incision was closed in a continuous interlocking manner with catgut 0. The peritoneal layer was also closed with catgut 2.0. Ensuring proper hemostasis, the abdomen was closed in layers. No vaginal septum was identified.

The postoperative course was uneventful. We advised her to perform an MRI scan for any associated renal anomaly, but she declined further imaging. The patient had an uneventful recovery and was discharged in stable condition on the fifth postoperative day. She was counseled on the uterine congenital abnormality and informed of the need for close follow-up with future pregnancies and the need for a multidisciplinary team during her labor and delivery.

## Discussion

Uterus didelphys is a rare congenital anomaly that results from the complete failure of fusion of the Müllerian ducts during embryological development, resulting in two separate uterine cavities and usually two cervices as well; in some cases, a longitudinal vaginal septum may be present [7,8].

Despite the anatomical abnormality, many women with uterus didelphys have normal reproductive capability. However, several studies have documented an increased risk of adverse obstetric outcomes including miscarriage, preterm birth, fetal malpresentation, and increased rates of cesarean section [9].

Heinonen reported that women with uterus didelphys often need cesarean section because of malpresentation or obstetric problems, although successful vaginal delivery is possible in selected cases [10,11]. Additionally, congenital anomalies of the uterine anatomy are associated with reproductive failure and pregnancy complications, and hence early diagnosis is important for appropriate obstetric management [12].

Diagnosis of uterus didelphys can be made using imaging tools such as three-dimensional ultrasonography, MRI, hysterosalpingography, or laparoscopy, which provide a detailed assessment of uterus anatomy [13,14]. However, routine antenatal ultrasound may not always detect uterine anomalies in the uterus, especially in resource-limited settings where advanced imaging techniques are not routinely available [15-18].

In the present case, the anomaly was not diagnosed during the antenatal period. Similar cases have been described in the literature where congenital anomalies of the uterus were only discovered during surgical procedures. Such findings add credence to the importance of careful assessment of uterine morphology on antenatal imaging.

Early identification of uterine anomalies is important because it allows closer antenatal surveillance, identification of obstetric risks, and appropriate delivery planning, which may help improve maternal and neonatal outcomes.

## Conclusions

Uterus didelphys is a very rare congenital anomaly of the Müllerian ducts that may remain undiagnosed until pregnancy or surgical intervention. Although fertility is usually maintained, the disease is associated with an increased risk of obstetric complications, including miscarriage, preterm labor, malpresentation, and cesarean section. This case raises awareness of the importance of careful evaluation of uterine anatomy during antenatal imaging and clinical awareness of congenital anomalies of the uterus, particularly in resource-limited settings where advanced diagnostic imaging may not always be available. Early diagnosis and proper obstetric management are vital in improving pregnancy outcomes. Finally, this case highlights the need for individualized care and monitoring of women with uterine anomalies, especially when unusual findings arise during delivery and even after delivery. Proper counseling and an experienced obstetrician, along with the presence of a neonatologist, are crucial in subsequent pregnancies.
